# The Destruction of Children’s Teeth–Cause and Prevention

**Published:** 1896-04

**Authors:** J. G. Henister

**Affiliations:** Baltimore, Md.


### THE DENTAL REGISTER.

Vol. L.] **APRIL, 1896.** [No. 4.

### COMMUNICATIONS.

### The Destruction of Children's Teeth—Cause and Prevention.

Read by title in the Section on Dental and Oral Surgery at the Forty-sixth Annual Meeting of the American Medical Association, at Baltimore, Md., May 7-10, 1895.

BY J. G. HENISTER, M.D., BALTIMORE, MD.

The destruction of children's teeth is to me, as it must be to all of you, a source of the greatest regret, and ordinary methods have failed to overcome the difficulty. It would be useless for me to attempt to go into details as to the cause of this condition, as it must be perfectly apparent to all who stop to consider a moment. I think the first and the greatest cause is neglect and ignorance as to the proper time and means of caring for the teeth. I tell you, gentlemen, it is astonishing to see the ignorance of some parents in regard to the teeth of their children. They will come into your office with a child and point to a six-year molar with the crown nearly destroyed, and say: "Doctor, I wish you would extract this little snag." You will answer, "That is a permanent tooth and should be preserved." They reply, "Oh no, that is only one of the baby teeth." You insist that it is a permanent tooth and refuse to extract it, and they leave your office with a look of great incredulity, go to some one else who will extract it, and you will probably never see them again. This ignorance is not confined to a few of the poor, who are unable to procure the services of a dentist, but extends to others who have plenty of time and means, but who never think of their children's teeth until they are crying with the pains of toothache. There is still another class of parents, who do all that they think necessary by insisting upon the use of the tooth-brush and dentifrices, but never think of consulting a dentist so long as the child does not complain. They make a mistake, common to a great many, that the incisors are the first permanent teeth, and thus utterly neglect the six-year molar, thereby leaving it for a year or two exposed to the inroads of caries, without any protection whatever.

Another important cause of this decay is in the use of improper foods; by double bolting in the craze for white flour and bread, the phosphates or bone-making properties are taken away, and, as a consequence, the teeth of children are imperfectly developed. I suggest to my patients the use of corn bread and graham bread, and I also prescribe the "Syrup of Hypophosphites" to endeavor to check and restore the loss of the lime salts of the teeth.

I have tried to enumerate a few of the causes of the loss of the teeth of children; I now come to the methods of saving them and to the responsibility of dentists and physicians alike. I have spoken to physicians about the matter, and they nearly all disclaim any responsibility, saying: "The teeth are not in our line, that is your work." This is a serious mistake. No physician who has the welfare of his patients at heart can afford to neglect the teeth, for in the mouth begins the process of digestion, and if the food is not properly masticated, it goes into the stomach in lumps, and gives the digestive organs there so much work that they become impaired and unable to properly perform their functions. We have been called a "nation of dyspeptics" and the cause has been attributed to the rush and hurry of American life, in eating too much and too rapidly, but I do not think this is the entire cause. The teeth being imperfect, imperfect mastication is the result, which is an important factor in this distressing complaint. The physician says, "What can I do? You would not have me fill the teeth?" No, that is entirely unnecessary and out of his line, but he can, at least, examine the teeth of children, and insist upon the parents taking them to some careful dentist.

This is not a mere suggestion; it is his duty, a sacred obligation. If the nose, eye or ear is affected, does he not examine it and insist upon the parent taking the child to a specialist? Do you not think that the teeth are of sufficient importance to warrant equal care? I think so, and say that the health of future generations will, in a great measure, depend upon the preservation of the teeth now. It has been predicted that the "coming man" will be toothless and hairless, and if things continue as they have been doing, this will probably be so. It has been demonstrated by scientists that deformities and malformations can be transmitted from parent to offspring. If it is so with other parts of the body, why not with the teeth? Jaws are sometimes entirely stripped of teeth, and when some teeth do remain they are only vestiges of what they should be. This I think is a deformity that can be transmitted.

I have observed on several occasions that peculiarities of the mouths of parents were inherited by children; for instance, I saw a mother who never had a left superior lateral incisor. One of her sons, in fact, the only one of her children I ever saw, had the same peculiarity. So I might go on giving examples of what will probably happen if we do not save the teeth of the children. You all know that the field of action of the dentist is, in a manner, circumscribed. He can not regularly go to the home of his patients to examine their teeth, but must wait until they come to him. Now here is where the physician should do his part. He usually has the care of the child from its very infancy, and if he would occasionally make a careful examination of the teeth of his little patients, and send them to the dentist, the small cavities of decay could be filled with some simple material, and the teeth thus preserved indefinitely. A short time ago a physician, whom I know, insisted that a mother should take her little girl to a dentist, saying the six-year molars were decayed and required filling. She was brought to me and I found that the teeth were badly decayed, and so sensitive that I had great difficulty in persuading the child to allow me to do anything to them. She was eleven years of age; if this physician had examined her mouth regularly, and insisted upon her going to a dentist the caries could not have made much progress. Dr. Bonwill, the eminent Philadelphia dentist, has said that the only way he has ever been able to do anything with the six-year molar was to fill every tiny spot of decay. So once again I beg of physicians to watch the mouths of the children and send them to a dentist.

Now, gentlemen of the dental profession, what is our duty? In the first place, we must endeavor to educate our patients. How can it be done? I have seen little treatises upon the care of the teeth, written from time to time by dentists and others, who have given this matter some attention, but as a rule they were too scientific for the average lay reader. What is needed, and what I would suggest, is the publication, under the auspices of the American Medical Association, or the National Association of Dental Faculties, of a small tract or leaflet, written in plain, simple language, so that any one can understand it, describing the order of the eruption of the permanent teeth, and the proper means of caring for them, and warning parents of the necessity of preserving the temporary teeth until the permanent teeth appear. These little papers could be given our patients, and I think, in this manner, great good could be accomplished. Another method that has occurred to me, is the preparation of short articles upon the care of the teeth, written in such a manuer as to interest, and at the same time instruct, the children as to the necessity of taking care of their teeth, these articles to be published in juvenile papers, such as *St. Nicholas*, *Chatterbox*, *Golden Days*, etc. This is only an idea of mine, and may not be practicable. Everything is to be published with the sanction of some recognized medical or dental society, thus removing from it every semblance of quackery or advertising.

Now, gentlemen, I have presented these few ideas of mine. I have attempted nothing scientific, and have given no new and startling theories, but have endeavored to express myself in plain, simple language; and I would ask you not to hesitate in criticism of me and my suggestions, for my object has been to provoke discussion, thereby bringing out the views of those present upon the subject. I thank you for your kind attention, and will say, in conclusion, if this paper is the means of saving only a few of the teeth now annually lost, I will be very happy.

